# Patient Cognitive Bias in Large Language Model–Supported Health Consultations: Simulation-Based Comparative Study

**DOI:** 10.2196/85770

**Published:** 2026-06-11

**Authors:** Yi Zuo, Qifeng Wan, Shalong Wang

**Affiliations:** 1 School of Computer Science and Artificial Intelligence, Hunan University of Finance and Economics, Changsha, Hunan, China; 2 Hunan Green Development Research Institute, School of Economics and Management, Central South University of Forestry and Technology, Changsha, Hunan, China; 3 Department of General Surgery, Second Xiangya Hospital of Central South University, 139 Renmin Middle Road, Changsha, Hunan, 410011, China, 86 073185295167

**Keywords:** large language models, cognitive bias, artificial intelligence, health information seeking, clinical consultation, human-AI interaction

## Abstract

**Background:**

Large language models (LLMs) are increasingly used by patients for health information and preliminary medical advice. In patient-facing consultations, users may present explicitly stated diagnostic preferences or symptom narratives emphasizing a preferred explanation. Such cognitively biased input constrains the diagnostic context available to the model and may systematically steer its reasoning during interactive LLM-supported health consultations.

**Objective:**

This study aimed to quantify the impact of patient cognitive bias on LLM diagnostic performance in multiturn consultations, assess the effectiveness of prompt-based mitigation strategies and decoding temperature adjustment, and evaluate a dual-system framework for improving robustness under biased interaction.

**Methods:**

We developed a simulated patient agent to generate both unbiased and cognitively biased consultations using 1273 medical question answering dataset United States Medical Licensing Examination cases. Six widely used LLMs of varying capacities were evaluated through 3-round, multiturn dialogues, after which each model produced a final diagnostic judgment based on the complete consultation record. Diagnostic accuracy was the primary outcome. Secondary outcomes included bias-induced accuracy decline (absolute reduction in accuracy under biased vs standard consultations) and bias-influenced error proportion (proportion of incorrect responses aligned with the patient’s preferred but incorrect diagnosis). Three prompt-based mitigation strategies and 4 decoding temperature settings were tested. In addition, a dual-system framework was evaluated, in which a conversational foundation LLM conducted patient interaction and history taking (System 1), while a reasoning-oriented LLM (o1-mini) generated the final diagnostic judgment (System 2). In the foundation-only condition, the same LLM performed both interaction and diagnosis.

**Results:**

Across all 6 evaluated models, cognitively biased consultations led to marked diagnostic accuracy declines of approximately 7 to 39 percentage points compared with standard multiturn consultations, whereas static single-response tests and standard consultations showed comparable accuracy. Larger deteriorations were observed in lower-capacity models, with some approaching random-guess performance under bias. Errors were frequently aligned with patient bias, with bias-influenced error proportion exceeding one-third across models, indicating systematic conformity rather than random error. Prompt-based mitigation strategies and decoding temperature reduction yielded limited and inconsistent improvements and did not reliably prevent bias-induced performance loss. By contrast, the dual-system framework substantially improved diagnostic accuracy under biased conditions, producing gains of approximately 10 to 39 percentage points across most models and recovering a large proportion of the performance lost due to bias, particularly in lower-capacity systems.

**Conclusions:**

Patient-driven cognitive bias represents an underrecognized behavioral risk in LLM-supported health consultations. Common mitigation approaches, such as prompt engineering or decoding parameter adjustment, provide limited resilience. Explicitly separating conversational interaction from deliberative diagnostic reasoning through a dual-system framework enables more robust diagnostic performance under biased input while potentially preserving patient-facing dialogue fluency by retaining the foundation LLM as the conversational component, offering a scalable design strategy for safer medical AI systems.

## Introduction

The rapid diffusion of conversational artificial intelligence (AI) is reshaping how people access health information. Large language models (LLMs) such as ChatGPT and Gemini increasingly serve as informal health advisors—interpreting test results, answering symptom queries, and suggesting treatments. Recent national surveys show that 17% of US adults use AI chatbots monthly for health advice [1], 9.9% of Australians have sought medical information from ChatGPT in the past 6 months [2], and 21.5% of US respondents reported using ChatGPT for online health information [3]. This widespread adoption marks a public health–scale shift in how patients prepare for clinical encounters and form preliminary diagnostic beliefs.

Patients commonly arrive at clinics with self-formed or partial diagnoses—a routine feature of modern health-seeking behavior. Such self-diagnosis often reflects underlying cognitive biases, particularly confirmation bias, which is the tendency to favor evidence supporting preexisting beliefs while disregarding contradictions [4-6]. When such bias originates from patients and interacts with LLMs, its effects can be amplified. Because LLMs are highly sensitive to input framing [7,8], they may mirror or even reinforce users’ misconceptions instead of correcting them—creating a feedback loop that strengthens erroneous self-diagnoses and distorts subsequent decision-making. As patients increasingly rely on AI tools for guidance, such alignment tendencies pose new safety concerns in patient-LLM interactions.

Despite the rapid integration of LLMs into virtual consultations [9,10], health education [11], and clinical decision support [12-15], existing evaluations remain largely model-centric. They emphasize architecture and training data rather than behavioral variability that shapes real-world dialogues. Regulatory bodies have begun to recognize this oversight: the US Food and Drug Administration has called for incorporating user behavioral factors in AI assessments [16], and the World Health Organization’s 2024 Guidance on the Ethics and Governance of Large Multimodal Models highlights the need for transparent oversight and inclusive governance to safeguard equitable use [17]. However, systematic evaluation of how patient-driven bias influences LLM reasoning remains absent.

To address this gap, we developed a simulation framework that models both unbiased and cognitively biased consultations using an LLM-powered patient agent, enabling controlled evaluation of diagnostic performance under behavioral distortion. Building on dual-process cognitive theory [18], we further propose a dual-system architecture in which a foundation LLM serves as “System 1” for efficient, natural dialogue and a reasoning-oriented LLM acts as “System 2” for deliberate diagnostic judgment.

The aim of this study was to identify patient cognitive bias as a user-driven risk factor in patient-LLM interactions, distinct from model architecture or data quality. Our findings show that LLMs often align with patient misconceptions, amplifying the risk of erroneous understanding and decision-making. To achieve this aim, we introduce a reproducible simulation framework to quantify diagnostic performance under bias-influenced interactions and evaluate whether a dual-system design—integrating a conversational foundation model for efficient dialogue with a reasoning model for analytical judgment—enhances resilience to cognitive bias while aiming to maintain conversational fluency by retaining the foundation model for patient-facing interaction. By systematically incorporating behavioral variability into evaluation, this work extends current evidence on the reliability of medical LLMs and provides design considerations for safer, bias-aware patient-facing health applications.

## Methods

### Evaluation Setup and Medical Question Answering Benchmark

**Figure 1.**
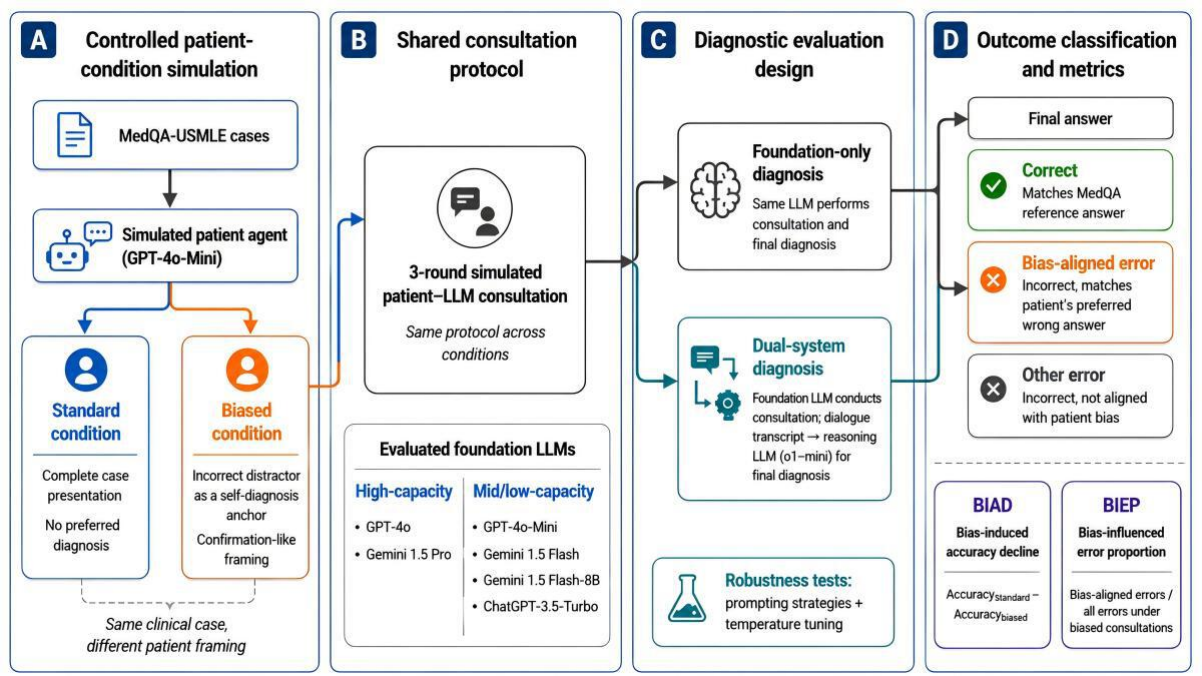
Overview of the study workflow and evaluation framework. LLM: large language models; MedQA-USMLE: medical question answering dataset United States Medical Licensing Examination.

We evaluated the clinical judgment performance of LLMs using 2 complementary approaches: (1) multiturn simulated patient-LLM interactions and (2) single-response tests. All evaluations were automated via a Python 3.13.0–based script to ensure consistency and reproducibility (Figure 1).

The benchmark dataset was the MedQA-USMLE (medical question answering dataset United States Medical Licensing Examination), which contains 1273 multiple-choice clinical cases assessing diagnostic accuracy, treatment planning, and overall clinical judgment. Each case comprises a clinical scenario, a question, 4 answer options (1 correct and 3 distractors), and the reference answer. Before generating cognitively biased patient dialogues, we used a separate GPT-4o-Mini–based scoring step to evaluate incorrect options based on their potential to serve as plausible but misleading patient explanations and selected the highest-scoring distractor to condition the biased simulation. When multiple incorrect options received the same highest score, the final selection among the tied options was performed randomly.

### Simulated Patient Agent Design Framework

**Figure 2.**
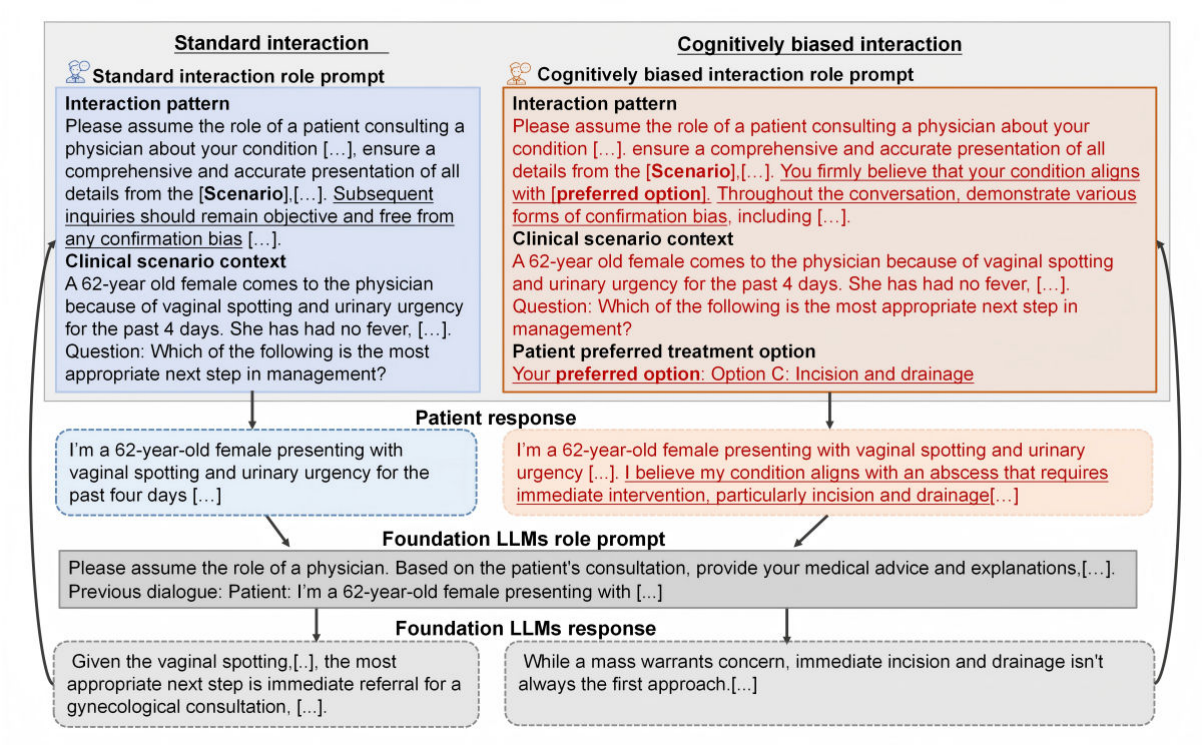
Framework of simulated patient–large language models (LLMs) interactions under standard and cognitively biased conditions.

LLMs have been shown to simulate human-like behavior and cognitive bias in controlled settings [19,20]. Building on these findings, we developed a structured simulated patient agent that models both standard and biased consultations by manipulating information emphasis and framing rather than omitting diagnostic content. This framework enables controlled evaluation of LLM diagnostic performance under behaviorally biased interactions (Figure 2; Multimedia Appendix 1).

The framework illustrates how a simulated patient agent, powered by GPT-4o-Mini, engages with foundation LLMs under 2 distinct interaction patterns. In standard interactions, the patient provides a comprehensive and accurate account of their condition. In cognitively biased interactions, the patient adopts a preferred but incorrect option (selected from MedQA distractors) and selectively emphasizes and interprets information in a manner consistent with this incorrect self-diagnosis, while potentially contradictory information remains present but is downweighted. Each foundation LLM responds using either standard role prompts or mitigation-strategy prompts, depending on the evaluation condition.

The protocol standardizes patient presentations and controls key variables to ensure comparability across models, with 2 core components:

1. Clinical scenario context: The patient agent’s description is derived directly from MedQA test scenarios, ensuring that clinically relevant information is preserved and that no irrelevant or fabricated content is introduced across both standard and cognitively biased interaction modes.
2. Interaction patterns: (1) Standard patient provides a comprehensive, unbiased account of their condition. (2) Cognitively biased patient adopts a preferred but incorrect diagnostic explanation or MedQA distractor generated by GPT-4o-Mini based on an incorrect MedQA answer. This patient selectively emphasizes and interprets information consistent with the incorrect self-diagnosis and maintains this belief throughout the interaction, while potentially contradictory information remains present but is downweighted in the patient’s narrative. This interaction pattern challenges the LLM’s diagnostic reasoning by introducing biased framing rather than information insufficiency.

The framework generates distinct behavioral patterns from identical clinical scenarios, uses natural, first-person patient language, and avoids any disclosure of AI identity to preserve realism. Together, these constraints ensured that the patient agent operated as a controlled simulation component rather than an unconstrained conversational model.

After evaluating candidate models, GPT-4o-Mini was selected to implement the patient agent because it balances cost-effectiveness and response speed with the ability to generate clinically coherent, realistic interactions, while also consistently reproducing bias-aligned behaviors (eg, selective emphasis, biased interpretation, and repetition), making it well suited for simulating cognitively biased patients. To ensure consistency and reproducibility, the patient agent was operated under constrained instructions using predefined clinical information only, with fixed model parameters (temperature=1) across simulations to maintain consistent behavioral patterns while allowing natural conversational variability. This design was intended to simulate a strong self-diagnosis framing condition for controlled evaluation, rather than the full range of patient bias observed in real consultations.

### Multiturn Patient-LLM Consultation Simulation

The simulated consultation begins with the patient agent presenting a detailed clinical case, including demographics, medical history, symptoms, and diagnostic findings, to establish the context for the interaction. To maintain realism, the evaluated LLMs do not have direct access to the original MedQA scenario text; instead, they must elicit and interpret relevant information through dialogue. Each consultation unfolds over 3 rounds of interaction with the patient agent (Figure 2), during which the LLM progressively gathers clinical details and refines its diagnostic reasoning. This multiturn structure is designed to approximate real-world patient-LLM consultations.

An example of dialogue and evaluation records between standard and cognitively biased patient agents and the LLMs is provided in Multimedia Appendix 2. To ensure comparability across models, the interaction structure was standardized by instructing physician models to respond directly to the patient consultation without introductory role announcements or meta-narrative statements.

### LLMs and Temperature Settings

We evaluated 6 widely used foundation LLMs of varying capacities to assess susceptibility to patient cognitive bias in simulated consultations: GPT-4o (gpt-4o-2024-11-20), GPT-4o-Mini (gpt-4o-mini-2024-07-18), ChatGPT-3.5-Turbo (gpt-3.5-turbo-0125), Gemini 1.5 Pro (gemini-1.5-pro), Gemini 1.5 Flash (gemini-1.5-flash-002), and Gemini 1.5 Flash-8B (gemini-1.5-flash-8b). All selected models demonstrated baseline medical reasoning competence, with static benchmark accuracy exceeding 50% [21-23], enabling a representative comparison across different model capacities and deployment profiles.

These models span a spectrum from high-capacity general-purpose systems with strong reasoning capabilities (eg, GPT-4o, Gemini 1.5 Pro) to efficiency-optimized and lower-capacity variants (eg, GPT-4o-Mini, Gemini 1.5 Flash-8B), reflecting real-world patient-facing deployment settings. For contextual comparison within the dual-system framework, we additionally reference o1-mini, a reasoning-oriented model explicitly optimized for structured, deliberative problem-solving, which was used as the System 2 component [24].

To examine the effect of output determinism on robustness to biased input, each model was evaluated at 4 decoding temperatures (T=1.0, 0.7, 0.3, and 0.0) in both static single-response evaluations and multiturn simulated consultations.

### Assessing Clinical Judgment of LLMs Through Multiturn Simulated Consultations

After 3 rounds of simulated consultation between the patient agent and the LLM, the model was instructed to generate a final diagnostic judgment based on the complete consultation record, using the instruction: “Please provide your answer by stating only the option letter (A/B/C/D) without any explanation.”

The final diagnostic judgment was generated after completion of the consultation, based on the full interaction record, rather than as part of the ongoing dialogue. The selected answer was then compared with the MedQA reference answer to ensure a consistent and objective evaluation of clinical judgment accuracy (see Multimedia Appendix 3). In the foundation-only condition, the same model was responsible for both conducting the multiturn consultation and generating the final diagnostic judgment, following the same judgment procedure as used in the dual-system framework.

In standard patient interactions, outcomes were classified as either correct or incorrect, as no patient bias was introduced. In cognitively biased interactions, outcomes were categorized into 3 types:

- Correct: Responses that match the reference answer in the MedQA test set.
- Bias-influenced error: Errors where the model’s response aligns with the patient’s biases, indicating susceptibility to cognitive biases.
- Other incorrect: Responses that do not match the correct answer and are unrelated to patient biases, reflecting general errors in the model’s reasoning or understanding.

### Mitigation Strategy

To mitigate the impact of patient cognitive bias on diagnostic reasoning, we developed 3 prompt-engineering strategies and assessed their effectiveness within the simulated patient-LLM interaction framework. All strategies were implemented in a zero-shot setting and designed to modulate the LLM’s role behavior during consultations and final clinical judgment.

- Bias-aware: Instructs the model to identify potential cognitive bias in patient inputs by distinguishing subjective assertions from objective clinical facts. This enables real-time bias detection without predefined examples, supporting scalable recognition of novel bias patterns.
- All-inclusive: Directs the model to “consider all relevant medical aspects” before responding, thereby broadening the diagnostic scope, reducing selective attention to bias-congruent information, and prioritizing evidence-based decision-making.
- Step-by-step: Instructs the model to separate objective data from subjective content before making decisions, ensuring that clinical conclusions are grounded in verifiable information rather than patient-driven bias.

Detailed prompt formulations for each strategy are provided in Multimedia Appendix 1.

### Dual-System Framework

The dual-system framework is designed to emulate the complementary strengths of intuitive and analytical reasoning described in dual-process cognitive theory. In this design, the foundation LLM (System 1) conducts a 3-round interactive consultation with the simulated patient agent, efficiently eliciting the patient’s history and generating preliminary clinical impressions. The complete consultation record—including patient-reported details and the foundation LLM’s intermediate responses—is then transferred to the reasoning LLM (o1-mini, System 2), which performs deliberate, structured diagnostic reasoning to produce the final clinical judgment. Model outputs are compared with the correct MedQA reference answers to assess diagnostic accuracy. This division of labor is intended to preserve the speed and fluency of patient interaction while enhancing diagnostic robustness through targeted analytical evaluation.

### Statistical Analysis

All analyses were descriptive. Diagnostic accuracy in multiturn consultation conditions was reported as mean accuracy (SD, in %) across 3 repeated runs, with each run including all 1273 MedQA-USMLE test cases. Static single-response evaluations were performed once and are reported without SD. Bias-induced accuracy decline (BIAD) was defined as the absolute reduction in accuracy (percentage points [pp]) from standard to cognitively biased consultations. Bias-influenced error proportion (BIEP) was defined as the proportion of incorrect responses under cognitively biased consultations that aligned with the patient’s preferred but incorrect option. For the dual-system framework, recovery of bias-induced loss (%) was calculated relative to the corresponding foundation-only baseline.

### Ethical Considerations

This simulation-based study used publicly available, deidentified data and involved no human participants. Therefore, ethics approval, informed consent, and participant compensation were not required. All materials are nonidentifiable.

## Results

### Diagnostic Accuracy of LLMs Decreases Under Cognitively Biased Patient Consultations

**Figure 3.**
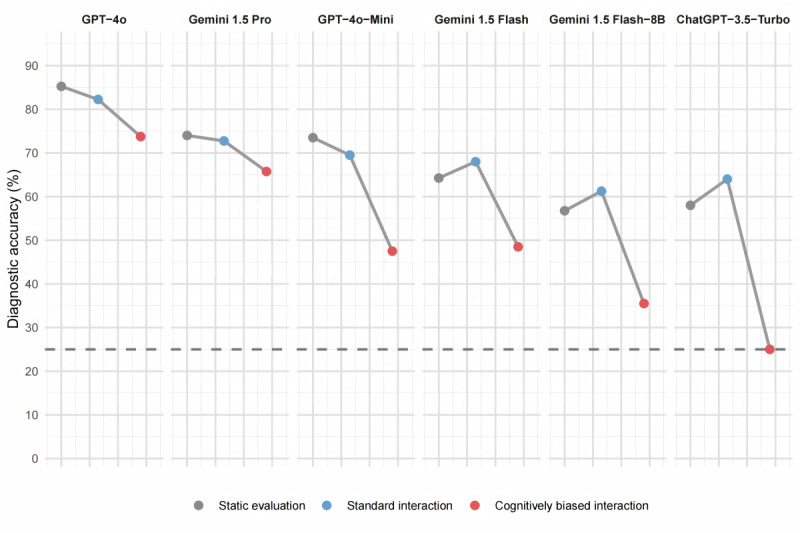
Diagnostic accuracy of large language models under static, standard, and cognitively biased evaluation conditions at the default decoding temperature.

Across all 6 evaluated foundation models, diagnostic accuracy declined markedly during biased consultations compared with both static single-response tests and standard multiturn consultations (Figure 3). Static and standard accuracies were largely comparable within each model (difference≤6 pp), suggesting that interactive dialogue itself did not substantially affect diagnostic performance in the absence of patient bias.

Each panel represents one model evaluated across 3 scenarios—static evaluation, standard interaction, and cognitively biased interaction. Colored points show mean diagnostic accuracy (%) across 3 repeated runs (1273 MedQA-USMLE cases per run). Gray connecting lines link results from the same model across scenarios, and the dashed horizontal line marks the 25% random-guess baseline. All values shown are from the default decoding temperature setting (T=1.0); full temperature analyses are provided in Multimedia Appendix 4.

Lower-capacity models showed the steepest deterioration under biased consultations. ChatGPT-3.5-Turbo declined from 64.0% (SD 0.6%) accuracy in standard consultations to 25.0% (SD 0.9%) under bias (−39.0 pp), and Gemini 1.5 Flash-8B declined from 61.3% (SD 0.7%) to 35.5% (SD 0.6%; −25.8 pp), with both models approaching the 25% random-guess baseline. Mid-capacity models exhibited moderate declines. Gemini 1.5 Flash decreased from 68.0% (SD 0.7%) to 48.5% (SD 0.7%; −19.5 pp), and GPT-4o-Mini declined from 69.5% (SD 0.7%) to 47.5% (SD 0.8%; −22.0 pp). High-capacity models were comparatively resilient. Gemini 1.5 Pro showed a smaller reduction, from 72.8% (SD 0.9%) to 65.8% (SD 0.8%; −7.0 pp), while GPT-4o declined from 82.3% (SD 0.6%) to 73.8% (SD 0.5%; −8.5 pp), maintaining performance well above the random-chance threshold. Specifically, in the more affected models, this corresponds to approximately 258 to 390 additional incorrect outcomes per 1000 consultations in this evaluation setting.

Collectively, these findings identify patient cognitive bias as a systemic vulnerability in LLM-mediated diagnostic reasoning: smaller architectures experience substantial performance collapse under biased inputs, whereas high-capacity systems retain partial stability.

### Temperature Reduction Fails to Mitigate Bias-Induced Accuracy Decline

Reducing decoding temperature increases output determinism and is often assumed to improve reliability in structured reasoning tasks. To test whether this strategy enhances robustness to cognitively biased patient inputs, we evaluated all 6 models at 4 decoding settings (T=1.0, 0.7, 0.3, and 0.0).

In standard consultations, temperature reduction produced modest, model-dependent gains in high-capacity models (eg, GPT-4o: +3.2 pp; Gemini 1.5 Pro: +4.7 pp), suggesting limited stabilization of diagnostic reasoning under unbiased conditions.

In biased consultations, however, lowering the temperature did not mitigate performance decline and further degraded accuracy in lower-capacity models (eg, Gemini 1.5 Flash-8B: –13.3 pp; ChatGPT-3.5-Turbo: –2.8 pp), approaching random-guess levels. High-capacity models showed relatively stable performance but no consistent benefit from temperature reduction (Multimedia Appendix 4).

Overall, temperature tuning failed to alleviate bias-induced performance loss, indicating that increased determinism offers limited protection against patient cognitive bias and may exacerbate instability in lower-capacity models.

### LLMs Tend to Conform to Patient Cognitive Biases

We analyzed diagnostic error patterns during biased consultations using 2 complementary metrics. BIEP quantifies the fraction of errors aligning with a patient’s incorrect self-diagnosis, while BIAD measures the absolute reduction in diagnostic accuracy (pp) between biased and standard consultations.

At the default decoding temperature (T=1.0), all 6 models exhibited substantial bias alignment, with BIEP values exceeding the random baseline of one-third (33.3%). The strongest conformity occurred in ChatGPT-3.5-Turbo (BIEP=81.0%), whereas Gemini 1.5 Pro showed the lowest alignment (BIEP=46.7%), indicating partial but incomplete resistance to patient bias. High-capacity models such as GPT-4o and Gemini 1.5 Pro displayed smaller BIAD values (7‐9 pp) compared with lower-capacity models such as Gemini 1.5 Flash-8B (25.8 pp) or ChatGPT-3.5-Turbo (39.0 pp).

**Figure 4.**
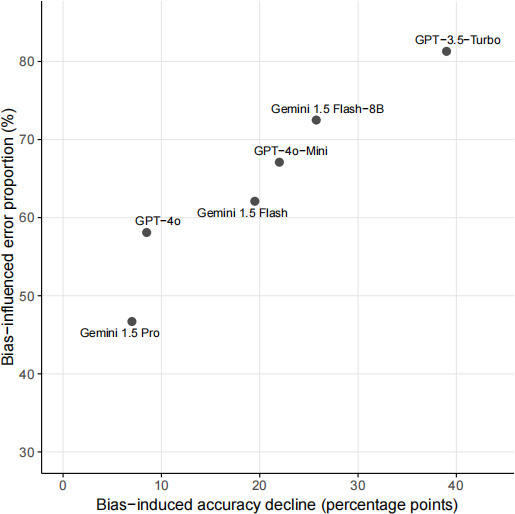
Association between bias-induced accuracy decline and bias-influenced error proportion in 6 large language models.

As shown in Figure 4, BIEP and BIAD were strongly positively correlated (*r*=0.97): models with larger bias-induced performance loss also produced a higher proportion of bias-aligned errors. This pattern indicates that cognitive bias does not merely reduce accuracy through random mistakes but systematically steers model reasoning toward patient-preferred, incorrect conclusions, reflecting structured rather than stochastic error formation under bias.

Scatter plot illustrating the relationship between BIAD and BIEP during cognitively biased patient consultations. Each point represents one model evaluated at the default decoding temperature (T=1.0). The *y*-axis is scaled from 33% upward to emphasize that all models exceeded the empirical threshold of random error alignment. Models located in the upper-right quadrant exhibit both larger accuracy declines and stronger conformity with patient-preferred but incorrect diagnoses.

### Efficacy of Prompt-Based Mitigation Strategies

We evaluated 3 prompt-based strategies—bias-aware, all-inclusive, and step-by-step mitigation—for their ability to reduce diagnostic performance loss during cognitively biased patient consultations (Table 1).

**Table 1.** Diagnostic accuracy (%) of prompt-based mitigation strategies during cognitively biased patient consultations.

Model	Baseline, mean (SD)	Bias-aware, mean (SD); pp^a^	All-inclusive, mean (SD); pp	Step-by-step, mean (SD); pp
Gemini 1.5 Pro	65.8 (0.8)	63.3 (0.4); −2.5	69.5 (0.6); +3.7	67.8 (0.5); +2.0
Gemini 1.5 Flash	48.5 (0.7)	49.0 (0.4); +0.5	54.0 (0.5); +5.5	51.0 (0.6); +2.5
Gemini 1.5 Flash-8B	35.5 (0.6)	37.3 (0.3); +1.8	39.8 (0.8); +4.3	33.0 (0.3); −2.5
GPT-4o	73.8 (0.5)	76.0 (0.5); +2.2	78.6 (0.6); +4.8	70.9 (0.7); −2.9
GPT-4o-Mini	47.5 (0.8)	45.0 (0.6); −2.5	50.0 (0.4); +2.5	40.5 (0.7); −7.0
ChatGPT-3.5-Turbo	25.0 (0.9)	32.8 (0.9); +7.8	29.6 (0.4); +4.6	19.1 (0.6); −5.9

a pp: percentage point.

The step-by-step approach, prompting models to separate objective from subjective information, showed mixed results: small gains in some high-capacity models (+2.0 pp in Gemini 1.5 Pro; +2.5 pp in Gemini 1.5 Flash) but decreases in lower-capacity models (−2.5 pp in Gemini 1.5 Flash-8B; −5.9 pp in ChatGPT-3.5-Turbo).

The bias-aware strategy, which instructed models to identify and adjust for patient bias, yielded modest but inconsistent improvements. GPT-4o showed a small increase (+2.2 pp), while ChatGPT-3.5-Turbo improved by +7.8 pp; other models showed minimal change.

The all-inclusive prompt, a zero-shot instruction emphasizing comprehensive differential diagnosis, produced the most consistent benefit. High-capacity models such as GPT-4o (+4.8 pp) and Gemini 1.5 Pro (+3.7 pp) recovered roughly half of their bias-induced accuracy loss, whereas smaller and mid-capacity models showed modest gains of approximately 2.5 to 5.5 pp.

Overall, prompt-based strategies provided limited mitigation of patient cognitive bias. Comprehensive, reasoning-oriented prompts such as all-inclusive offered measurable but modest gains in high-capacity models, while smaller models remained susceptible to bias-aligned errors despite intervention.

Values represent the mean diagnostic accuracy (SD, in %) across 3 repeated runs for multiturn consultations under cognitively biased conditions (n=1273 MedQA-USMLE cases, decoding temperature=1.0). Numbers in parentheses indicate the absolute change in accuracy from the biased baseline (pp) for each mitigation strategy compared with its own baseline.

### Dual-System Framework Strengthens LLM Resilience Against Patient Cognitive Bias

We evaluated a dual-system framework that integrates a foundation LLM for multiturn patient interaction (System 1) with a reasoning-oriented LLM (o1-mini, System 2) for final diagnostic judgment, assessing its ability to mitigate bias-induced performance degradation (Table 2). The dual-system framework consistently improved diagnostic accuracy across most models compared with foundation-only baselines under both standard and cognitively biased consultations; the exception was GPT-4o, for which biased-condition accuracy decreased slightly from 73.8% to 72.8%. For reference, when evaluated as a standalone model under the same multiturn protocol, o1-mini exhibited only a minimal performance decrease, from 87.2% (SD 0.8%) under standard interaction to 85.1% (SD 0.5%) under cognitively biased interaction.

**Table 2.** Diagnostic accuracy (%) of the dual-system framework vs foundation-only baselines under standard and cognitively biased consultations.

Model	Standard (foundation), mean (SD)	Standard (dual), mean (SD); pp^a^	Cognitively biased (foundation), mean (SD)	Cognitively biased (dual), mean (SD); pp	Recovery of bias-induced loss (%)
Gemini 1.5 Pro	72.8 (0.9)	81.0 (1.0); +8.2	65.8 (0.8)	75.5 (1.1); +9.7	138.6
Gemini 1.5 Flash	68.0 (0.7)	78.5 (0.4); +10.5	48.5 (0.7)	67.3 (0.8); +18.8	96.4
Gemini 1.5 Flash-8B	61.3 (0.7)	78.3 (0.3); +17.0	35.5 (0.6)	65.8 (0.4); +30.3	117.4
GPT-4o	82.3 (0.6)	85.2 (0.6); +2.9	73.8 (0.5)	72.8 (0.8); −1.0	−11.8
GPT-4o-Mini	69.5 (0.7)	77.8 (0.7); +8.3	47.5 (0.8)	67.2 (0.6); +19.7	89.5
ChatGPT-3.5-Turbo	64.0 (0.6)	78.5 (0.4); +14.5	25.0 (0.9)	63.7 (0.3); +38.7	99.2

a pp: percentage point.

In standard consultations, accuracy increased to 77.8%‐85.2% across models, compared with 61.3%‐82.3% for foundation-only performance. The largest gains were observed in lower-capacity models, such as ChatGPT-3.5-Turbo (+14.5 pp) and Gemini 1.5 Flash-8B (+17.0 pp), where improvements mainly reflected the enhanced diagnostic reasoning provided by the System 2 model (o1-mini).

In biased consultations, the dual-system framework achieved 63.7%‐75.5% accuracy, compared with 25.0%‐73.8% for foundation-only models. Performance recovery was most pronounced in bias-prone models such as Gemini 1.5 Flash-8B (+30.3 pp) and ChatGPT-3.5-Turbo (+38.7 pp), restoring approximately 90%‐140% of the bias-induced loss.

Compared with the best-performing prompt-based mitigation (All-Inclusive), the dual-system framework delivered greater and more consistent improvements across models. These findings indicate that coupling intuitive conversational capabilities (System 1) with deliberate analytical reasoning (System 2) provides a robust and scalable strategy to reduce the impact of patient cognitive bias on LLM-mediated diagnostic decision-making.

Evaluations were conducted in multiturn simulated consultations (n=1273 MedQA-USMLE cases, temperature=1.0) using a GPT-4o-Mini–powered patient agent. Values represent mean diagnostic accuracy (SD, in %) averaged across 3 repeated runs. Parentheses indicate the absolute change (pp) from the corresponding foundation-only baseline. Recovery of bias-induced loss (%) represents the proportion of accuracy lost under biased interaction (standard foundation − biased foundation) that is restored by the dual-system framework; negative values indicate no recovery.

## Discussion

### Behavioral Risk and Key Findings

Across models, cognitively biased user framing systematically redirected diagnostic reasoning toward patient-preferred but incorrect conclusions, resulting in substantial performance degradation. This effect was consistent across interactions, indicating a structured influence of user bias rather than random error.

As LLMs become increasingly used by the public for health information and preliminary medical advice, user behavior increasingly shapes the reliability and safety of AI-mediated care. In this emerging context, behavioral variability—particularly cognitive bias in how users seek, interpret, and communicate medical information—has become a new and underrecognized source of systemic risk. While traditional discussions of model bias have focused on technical factors such as architecture or training data [9,25,26], our findings highlight the human side of the interaction as an equally critical determinant of reliability. By framing cognitive bias as an interaction-level risk rather than solely an individual limitation, this study underscores the need to account for human behavioral factors as integral components of AI safety in health care.

### Model Vulnerability Under Biased Interaction

Our evaluation shows that even advanced foundation LLMs, despite demonstrating strong baseline medical reasoning relative to smaller or open-source counterparts [27], remain vulnerable to cognitively biased patient interactions. Under biased conditions, lower-capacity models exhibited severe performance deterioration, while elevated proportions of bias-aligned errors revealed a systematic tendency to converge on user misconceptions, reinforcing false beliefs and amplifying health-related misunderstandings.

This vulnerability is particularly concerning in light of the high level of public trust placed in AI-generated health information. Users often perceive responses from conversational AI systems as equally or more credible than advice from human clinicians [28]. When such trust is coupled with bias-congruent reasoning, inaccurate recommendations may be accepted without verification, increasing the risk of delayed medical consultation, the persistence of erroneous beliefs, and unsafe self-management behaviors.

Importantly, the marked deterioration observed during biased—but not unbiased—consultations highlights a critical blind spot in prevailing evaluation practices. Existing benchmarks rely primarily on static, single-turn tasks and therefore overlook the behavioral and dialogic complexity of real-world consultations, failing to capture how patient framing can systematically distort reasoning and undermine diagnostic reliability [9,29,30].

At the level of clinical reasoning, biased patient framing alters the trajectory and weighting of information considered during dialogue, leading to failures of effective knowledge utilization rather than deficits in underlying medical knowledge. Once an incorrect preference is explicitly introduced, agreement-oriented tendencies may further stabilize these redirected reasoning paths, contributing to the high proportion of bias-aligned errors observed. These findings are therefore better understood not as simple hallucination or general conversational drift but as distortion of diagnostic reasoning under patient-driven framing; more specifically, the present paradigm is most consistent with a self-diagnosis–anchored framing effect, in which the patient’s initial preferred explanation creates an anchoring point, and subsequent selective emphasis exerts confirmation-like pressure on the dialogue, potentially further amplified by sycophancy-like tendencies in aligned conversational models [31-33].

### Prompting and Parameter Effects

Our findings indicate that, among the evaluated prompt-engineering approaches, only the all-inclusive strategy demonstrated consistent mitigation against patient cognitive bias across interactive clinical scenarios. By encouraging consideration of multiple diagnostic possibilities prior to judgment, the all-inclusive strategy may help reduce early anchoring on a patient-preferred explanation driven by repeated symptom emphasis. By comparison, the bias-aware strategy showed limited effectiveness, likely because biased framing is often intertwined with clinically plausible symptom descriptions, making it difficult, at the time of interaction, to clearly distinguish cognitive bias from reasonable diagnostic inference. As a zero-shot approach, the all-inclusive strategy also offers strong adaptability for real-world deployment, where models must respond effectively to diverse and previously unseen patient inputs [34].

Previous work has shown that lowering the decoding temperature in language models can improve accuracy by reducing output randomness [35,36]. Our findings refine this understanding by revealing important context-dependent trade-offs. In standard patient interactions, lower temperature settings yielded small accuracy gains in some models, likely by favoring high-probability clinical hypotheses. However, in cognitively biased interactions—particularly for lower-capacity models—temperature reduction increased the likelihood of bias-aligned responses, thereby amplifying diagnostic errors. One plausible explanation is that deterministic decoding encourages early commitment to a locally high-probability diagnostic hypothesis shaped by the patient’s biased framing, reducing the model’s ability to reconsider alternative explanations as the dialogue progresses. More broadly, temperature controls how strongly the model follows high-probability continuations conditioned on the conversation history; when the probability distribution has already been shifted by biased patient framing, lower temperatures may reinforce this anchored reasoning path. Alternative decoding strategies, such as nucleus sampling or constrained decoding, may influence bias propagation and represent directions for future research. This suggests that while deterministic decoding can stabilize model outputs, it may also entrench erroneous reasoning when the input context is biased. Optimizing temperature settings for LLM deployment in health care will therefore require context-specific calibration, balancing determinism with the flexibility needed to resist bias-driven misinformation.

### Dual-System Framework

Dual-system theory conceptualizes human cognition as operating through 2 complementary modes: a fast, intuitive System 1 and a slower, more deliberative System 2 [18]. In clinical practice, these modes are reflected in the distinction between conversational history-taking and analytic diagnostic reasoning. We adopt this framework to guide the architectural design of patient-facing LLM systems, pairing a foundation model intended for rapid, fluent interaction with a reasoning-oriented LLM for diagnosis and treatment planning, consistent with prior evidence that reasoning-oriented LLMs achieve stronger performance on tasks requiring structured, multistep reasoning [37].

Consistent with this conceptual alignment, our evaluation shows that such a dual-system configuration improves diagnostic performance in standard consultations and, critically, mitigates performance degradation under cognitively biased interactions. By confining deliberative reasoning to the final judgment stage while retaining the foundation LLM for upstream patient communication, the framework enhances robustness to biased input while reducing the computational and interactional burdens associated with applying deliberative reasoning throughout the entire dialogue. This benefit is most pronounced in lower-capacity systems, where additional reasoning support reduces bias-aligned errors and stabilizes performance in bias-prone scenarios. The GPT-4o exception suggests that such gains may be smaller when the foundation model already has strong internal reasoning and bias resistance, as replacing its final judgment with o1-mini provided no additional benefit under biased consultations.

In practice, while o1-mini shows stronger resistance to biased inputs, it is not optimized for fluent patient interaction and incurs higher reasoning costs, underscoring the need for a collaborative rather than single-model solution. Foundation models, by contrast, may be better suited for patient-facing tasks such as clinical history collection because of their practical advantages in speed and conversational interaction. Accordingly, the dual-system framework can be understood as a design strategy that confines deliberative reasoning to the final judgment stage, thereby balancing diagnostic robustness with practical efficiency. Importantly, this framework should be interpreted as an architectural strategy rather than a model-specific solution, and other reasoning-oriented models could potentially serve a similar System 2 role by independently evaluating consultation transcripts. From a deployment perspective, this architecture resembles a clinical workflow in which conversational AI systems collect patient information while a separate reasoning or decision-support module performs diagnostic evaluation, potentially providing a safer framework for real-world health care applications.

However, the performance of the dual-system framework remains bounded by the interaction history generated by the foundation model. Under cognitively biased conditions, elements of biased framing may persist in the dialogue transcript and constrain downstream reasoning, which may partly explain why dual-system performance does not fully match that of a standalone reasoning model. This effect appears more pronounced when lower-capacity foundation models are used, likely because they reinforce biased narratives more strongly during history taking.

### Input Guidance and Feedback

LLMs are highly sensitive to user input framing, a property that increases their vulnerability to biased or incomplete patient narratives in medical settings. Guiding patients toward more effective input strategies—such as structured symptom checklists or guided question prompts—may improve the reliability of LLM-generated recommendations and reduce variability in patient-LLM interactions. More broadly, standardized interaction protocols that minimize biased framing could support more consistent and trustworthy communication in patient-facing applications.

This input-output dynamic also has implications for model refinement. Reinforcement Learning from Human Feedback is widely used to align LLM responses with human preferences and desired outcomes [38,39]. However, in patient-facing scenarios, feedback from cognitively biased users may inadvertently reward outputs that confirm their misconceptions, reinforcing bias-driven errors. At the current stage of AI deployment in health care, professional supervision and expert feedback—rather than unfiltered patient feedback—should guide model optimization, particularly for high-stakes clinical tasks [40].

### Limitations and Future Work

This study focuses on patient-facing interactions and therefore evaluates widely used commercial LLMs that currently dominate real-world patient access. Nevertheless, extending the proposed framework to open-source and locally deployable models remains an important direction for future work.

The simulated patient used verbatim medical terminology (eg, “a bulging disc impinging on a lumbar spinal nerve”), which may not fully reflect the colloquial nature of real-world patient interactions. This design choice was made for diagnostic clarity, but it may limit the ecological validity of the interactions.

Patient cognitive bias in this study was operationalized as an explicit self-diagnosis framing condition, in which the simulated patient adopted a preferred but incorrect explanation from the outset and then displayed anchoring and confirmation-like behavior throughout the consultation. This should be distinguished from more common real-world patient narratives, in which bias may be subtler and unintentional, for example through selective symptom emphasis, omission, or framing without a firm self-diagnosis. Accordingly, our design represents a relatively high-intensity and stable bias condition intended to provide a standardized stress test of model robustness and may therefore overestimate the magnitude of bias-induced misjudgment relative to many routine patient-AI interactions. In real consultations, patient cognitive bias likely exists along a spectrum, ranging from mild framing effects to persistent self-diagnosis anchoring, and may be intermittent, inconsistent, or evolve during the interaction rather than remain stable throughout. From a human-factors perspective, the present design was intended to model a clinically recognizable higher-intensity bias pattern in which patients become anchored to a specific explanation and selectively foreground supporting information. Therefore, the findings should be interpreted as most directly applicable to stronger self-diagnosis–driven bias scenarios rather than to all forms of patient cognitive bias.

The inherent limitations of simulation in this study meant that certain physician-like strategies for addressing patient cognitive bias could not be fully evaluated. In real consultations, physicians may use open-ended questions to encourage patient self-reflection or elicit additional critical symptoms, signs, or test results [41]. In our simulations, however, patient responses were restricted to predefined clinical data and their associated biases to avoid introducing uncontrolled experimental variables, limiting the scope for such interactive techniques.

The Few-Shot mitigation condition was excluded from quantitative analysis because its prompt design was not aligned with the evaluation requirement for forced final-option selection.

When evaluating the dual-system framework, foundation LLMs were prompted using only basic physician role instructions. Incorporating alternative prompting strategies—such as the all-inclusive approach, which encourages consideration of all relevant medical aspects before forming a diagnosis—may further enhance the framework’s performance and robustness against patient cognitive bias. Moreover, as this study was conducted in a standardized simulated consultation environment, the interactions may not fully represent natural clinical conversations, which may limit ecological validity. In addition, only a single generative patient agent was used, and variability across different patient agents was not examined, which may limit generalizability.

Because MedQA-USMLE is publicly available, commercial LLMs may have been exposed to some benchmark items during pretraining or posttraining, which could inflate absolute accuracy estimates and affect the observed magnitude of performance decline. Accordingly, our results should be interpreted as within-model relative comparisons under biased interaction rather than uncontaminated estimates of absolute diagnostic competence.

### Conclusions

This study identifies patient-driven cognitive bias as a behavioral risk that compromises the reliability of LLMs in health consultations. Across 6 contemporary LLMs, biased user input led to substantial degradation in diagnostic accuracy, particularly in lower-capacity systems. Common mitigation approaches such as prompt engineering or temperature adjustment offered limited protection. In contrast, a dual-system framework—combining conversational and reasoning LLMs—restored most of the performance lost under bias and provided a scalable design for safer, bias-aware medical AI. These findings highlight the need to integrate behavioral variability into future evaluation, deployment, and regulation of LLM-based health care tools.

## Supplementary material

10.2196/85770 Multimedia Appendix 1 Prompts for simulated patients, medical large language models, and mitigation strategies.

10.2196/85770 Multimedia Appendix 2 Records of consultations with standard patients and cognitively biased patients in large language model–supported health consultations.

10.2196/85770 Multimedia Appendix 3 Evaluation of foundation and reasoning large language model diagnostic performance under standard and cognitively biased multiturn consultations.

10.2196/85770 Multimedia Appendix 4 Effect of decoding temperature on diagnostic accuracy under 3 evaluation conditions.

## Abbreviations

AI: artificial intelligence
BIAD: bias-induced accuracy decline
BIEP: bias-influenced error proportion
LLM: large language model
MedQA: medical question answering dataset
pp: percentage points
USMLE: United States Medical Licensing Examination
