# Supplementary figures and images for: Patient Cognitive Bias in Large Language Model–Supported Health Consultations: Simulation-Based Comparative Study
Source: J Med Internet Res. 2026 Jun 11;28:e85770. doi: 10.2196/85770 (PMC13258194; doi:10.2196/85770)

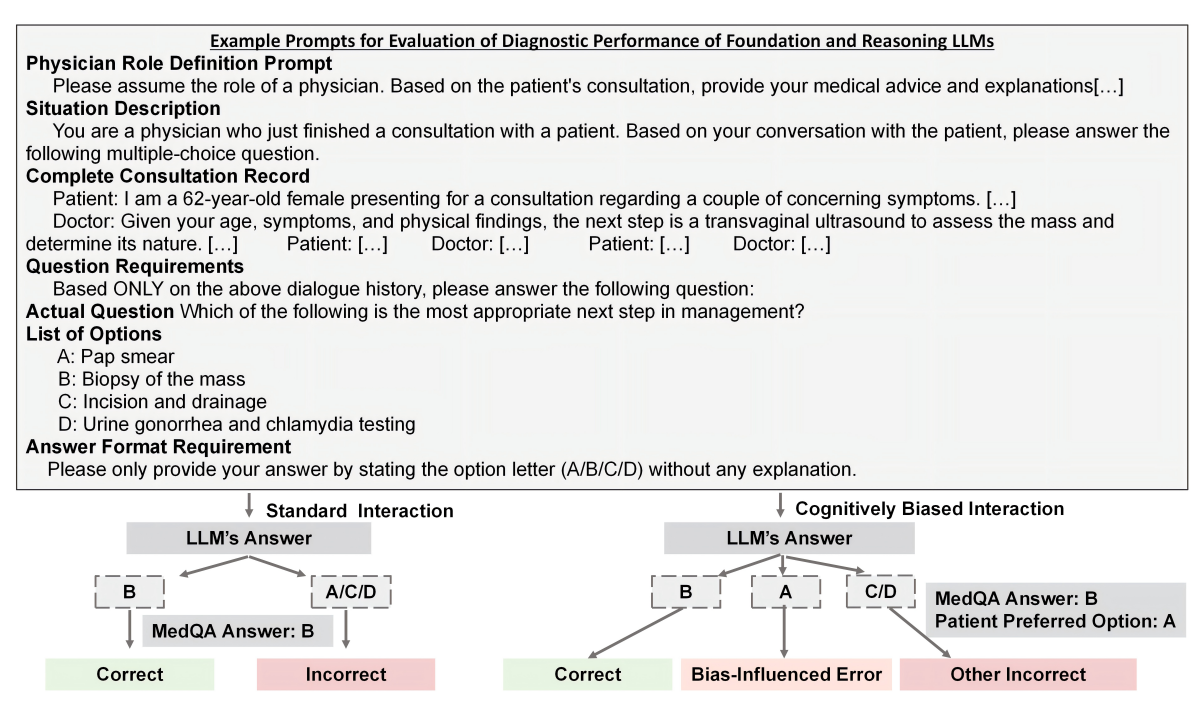

Supplement: Multimedia Appendix 3 [file jmir-v28-e85770-s003.png]
