# Supplementary material for: Patient Cognitive Bias in Large Language Model–Supported Health Consultations: Simulation-Based Comparative Study
Source: J Med Internet Res. 2026 Jun 11;28:e85770. doi: 10.2196/85770 (PMC13258194; doi:10.2196/85770)
Supplement: Multimedia Appendix 4 [file jmir-v28-e85770-s004.docx]

**Supplementary Table S1. Effect of decoding temperature on diagnostic accuracy under three evaluation conditions (Static, Standard, and Cognitively Biased)**

| **Model** | **Static T1.0** | **Static T0.7** | **Static T0.3** | **Static T0.0** | **Standard T1.0** | **Standard T0.7** | **Standard T0.3** | **Standard T0.0** | **Cognitively Biased T1.0** | **Cognitively Biased T0.7** | **Cognitively Biased T0.3** | **Cognitively Biased T0.0** |
| --- | --- | --- | --- | --- | --- | --- | --- | --- | --- | --- | --- | --- |
| Gemini 1.5 Pro | 74.0 | 71.0 | 73.8 | 73.0 | 72.8 (0.9) | 75.8 (0.4) | 76.8 (0.6) | 77.5 (0.5) | 65.8 (0.8) | 65.5 (0.4) | 62.5 (0.6) | 65.3 (0.5) |
| Gemini 1.5 Flash | 64.3 | 64.3 | 64.5 | 64.2 | 68.0 (0.7) | 70.3 (0.6) | 67.8 (0.5) | 69.3 (0.4) | 48.5 (0.7) | 52.5 (0.5) | 48.3 (0.4) | 47.5 (0.2) |
| Gemini 1.5 Flash-8B | 56.8 | 56.3 | 56.5 | 56.0 | 61.3 (0.7) | 56.5 (0.4) | 61.3 (0.6) | 61.5 (0.5) | 35.5 (0.6) | 32.8 (0.6) | 32.5 (0.2) | 22.2 (0.6) |
| GPT-4o | 85.3 | 86.5 | 87.0 | 87.2 | 82.3 (0.6) | 83.8 (0.5) | 84.3 (0.6) | 85.5 (0.4) | 73.8 (0.5) | 73.8 (0.4) | 72.5 (0.6) | 73.0 (0.5) |
| GPT-4o-Mini | 73.5 | 73.5 | 73.3 | 74.0 | 69.5 (0.7) | 71.0 (0.5) | 71.5 (0.4) | 71.2 (0.3) | 47.5 (0.8) | 43.8 (0.6) | 44.3 (0.5) | 47.4 (0.4) |
| ChatGPT-3.5-Turbo | 58.0 | 57.3 | 57.3 | 57.8 | 64.0 (0.6) | 62.5 (0.6) | 63.3 (0.4) | 63.5 (0.5) | 25.0 (0.9) | 23.8 (0.7) | 22.3 (0.4) | 22.2 (0.6) |

Values are diagnostic accuracy (%) across 1,273 MedQA-USMLE test cases. Standard and Cognitively Biased conditions are reported as mean (SD) across three repeated runs; Static evaluations were performed once and are reported without SD. Standard refers to unbiased three-round consultations, and Cognitively Biased refers to three-round consultations influenced by patient cognitive bias. Temperatures correspond to decoding settings.

Abbreviations: Static = single-response test; Standard = unbiased multi-turn consultation; Cognitively Biased = consultation influenced by patient bias.
